# Design of a High-Power, High-Efficiency GaN Power Amplifier for W-Band Applications

**DOI:** 10.3390/mi16090985

**Published:** 2025-08-28

**Authors:** Shuai Liu, Xiaohua Ma, Yi Zhang, Chunliang Xu

**Affiliations:** 1 School of Microelectronics, Xidian University, Xi’an 710071, China; xhma@xidian.edu.cn; 2 Microwave Applications Research Institute, The 13th Research Institute of China Electronics Technology Group Corporation, Shijiazhuang 050051, China; zhangyi1026@foxmail.com (Y.Z.); mrxcl@163.com (C.X.)

**Keywords:** W-band, GaN HEMT, high-efficiency power amplifier, balanced architecture, lange coupler

## Abstract

This paper presents a W-band high-efficiency and high-output-power power amplifier (PA) based on a 130 nm AlGaN/GaN-on-SiC HEMT process. The PA is designed to deliver optimal output power and gain performance across the entire W-band. A balanced architecture is adopted, combining two amplifier units through Lange couplers. High- and low-impedance microstrip lines are employed for input, output, and inter-stage matching. Each amplifier core adopts a three-stage configuration with gate width ratios of 1:2:4 to enhance gain. The bias network incorporates MIM capacitors and thin-film resistors to improve stability. Measured results indicate a small signal gain exceeding 17 dB under a gate voltage of −2.2 V and a drain voltage of +20 V. Within the 80–86 GHz frequency range, the PA achieves an output power above 34 dBm with a 22 dBm input power, corresponding to a power gain above 12 dB and a power-added efficiency (PAE) greater than 20%. The chip occupies a compact area of 2.65 mm × 3.75 mm. Compared with previously reported works, the proposed PA demonstrates the highest PAE within the 80–86 GHz band.

## 1. Introduction

Millimeter-wave power amplifiers (PAs) remain indispensable components in wireless communication systems, phased array radar systems, and imaging applications. Due to their low loss and high output power capabilities, Gallium Nitride (GaN)-based device technologies have attracted increasing attention [1,2,3,4]. Initially developed for lower frequency bands such as L-band and S-band, GaN technology has rapidly advanced into the microwave and millimeter-wave spectrum. GaN-based monolithic microwave integrated circuit (MMIC) PAs have continuously increased in operating frequency, along with reductions in device feature size, higher output power, and trends toward ultra-wideband operation and high efficiency [5]. These advancements make GaN a promising candidate for millimeter-wave applications and a mainstream technique for W-band solid-state power amplifiers.

For high-frequency applications, W-band (75–110 GHz) has seen considerable development. In 2006, M. Micovic et al. at HRL Laboratories reported the first GaN-based W-band PA [6]. Subsequent developments at HRL included a PA delivering 842 mW output power at 88 GHz with 14.8% PAE in 2010 [7], and a 2.138 W output PA with 19% PAE at 93.5 GHz in 2012 [8]. Following this, researchers around the world advanced the field further. James Schellenberg et al. demonstrated a broadband W-band PA utilizing on-chip traveling-wave power combining, achieving over 2 W continuous-wave output in the 75–100 GHz band, with a 3 W peak continuous-wave (CW) output and 3.6 W in pulsed mode [9]. Diego Marti et al. implemented a two-stage amplifier using a 100 nm GaN process, achieving 18.3 dBm saturated output power, 8.2 dB gain, and return losses better than 10 dB at 94 GHz [10]. In 2016, Shaobing Wu et al. introduced a three-stage amplifier in the 90–97 GHz range using a 100 nm AlGaN/GaN process, delivering 1.66 W output power, 21% PAE, and 13.7 dB power gain [11]. In 2017, E. Ture et al. designed a four-stage PA using a 100 nm 3D gate GaN HEMT process, which outperformed conventional GaN HEMTs by leveraging the superior device and circuit characteristics of the 3D gate structure. Their amplifier achieved over 1 W saturated output power, 12 dB power gain, and 6–8% PAE in the 86–94 GHz range under large-signal conditions [12]. In 2018, Maciej Cwiklinski et al. developed two full W-band PAs—one with three-stage and the other with four-stage cascade architecture—using a newly proposed wideband fan-shaped capacitor. These chips achieved output powers of 25.6–27.2 dBm and 27–28.6 dBm, and PAEs of 6.5–8.5% and 6.1–8.6%, respectively, across 70–110 GHz [13].

This paper presents a high-power W-band MMIC PA operating in the 80–86 GHz frequency range. The PA is fabricated using a 130 nm GaN T-gate high electron mobility transistor (HEMT) process and adopts a three-stage balanced topology. Lange couplers are used at the input and output stages for power splitting and combining between two amplifier paths. High- and low-impedance microstrip lines are used for input, output, and inter-stage impedance matching, and quarter-wavelength transmission lines are employed in the DC bias networks. The fabricated PA achieves over 21% PAE, greater than 12 dB power gain, and more than 3 W saturated output power within the operating band, with a total current consumption below 700 mA at a drain voltage of 18 V. The proposed PA features high output power, high efficiency, and excellent linearity with a compact layout. To the best of our knowledge, this is the first W-band GaN PA achieving over 3 W output power and more than 21% PAE.

The remainder of this paper is organized as follows: Section 2 introduces the device characteristics of the 130 nm GaN HEMT process. Section 3 describes the circuit architecture and design details. Section 4 presents the small- and large-signal measurement results of the fabricated PA and compares its performance with state-of-the-art W-band GaN MMIC Pas.

## 2. Device Technology

**Figure 1:**
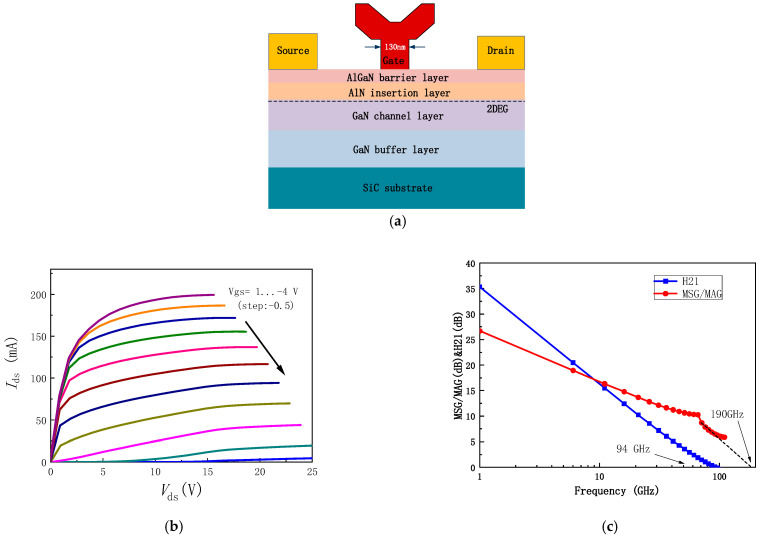
130 nm GaN-SiC Field Plate HEMT. (**a**) Schematic Structure of the Transistor; (**b**) I-V Characteristics; (**c**) Maximum Stable Gain (MSG), Maximum Available Gain (MAG), and Current Gain (H_21_) for a 4 × 37.5 μm HEMT.

The power amplifier designed in this work utilizes a 130 nm gate length GaN power MMIC process. Figure 1a shows the cross-sectional view of the 130 nm GaN HEMT process. The epitaxial layers are grown on a 4H-SiC substrate using metal–organic chemical vapor deposition (MOCVD). The epitaxial structure consists of a GaN buffer layer, a GaN channel layer, an AlN insertion layer, an AlGaN barrier layer, and a cap layer. To reduce interface and contact resistances, a thin AlN interlayer and Si-implanted ohmic contacts are introduced into the AlGaN/GaN heterostructure. A Y-shaped gate with a length of 130 nm is formed in the source-drain region using electron beam lithography.

After the transistor, microstrip lines, resistors, and capacitors are fabricated, the wafer is thinned to 50 μm, followed by gold plating on the backside. A thinner substrate not only improves thermal dissipation by lowering thermal resistance, but also mitigates via-hole inductance, which becomes particularly critical at W-band frequencies. Through-hole grounding is then achieved using reactive ion etching (RIE) technology. The thinner substrate not only provides a lower thermal resistance but also reduces the parasitic inductance of the through-holes. The optimized metal-insulator-metal (MIM) nitride deposition process helps minimize parasitic capacitance. The process includes two metal layers, with MIM capacitors having a capacitance of 300 pF/mm^2^ and thin-film resistors (TFRs) with a resistance of 50 Ω/□. The DC characteristics of the HEMT device are as follows: the maximum current (Imax) is 1.3 A/mm, and the maximum transconductance (Gmax) is 450 mS/mm. Figure 1b shows the I-V curve for a 4 × 37.5 μm HEMT device. The small-signal S-parameters of the HEMT are extracted on the wafer, yielding the short-circuit current gain (h21), maximum stable gain (MSG), and maximum available gain (MAG), as shown in Figure 1c. From the figure, the device’s cutoff frequency (*f*t) is 94 GHz, and the maximum oscillation frequency (*f*max) is 190 GHz. Additionally, the source-drain breakdown voltage exceeds 40 V, with a power density of approximately 3.81 W/mm.

## 3. Circuit Design

**Figure 2:**
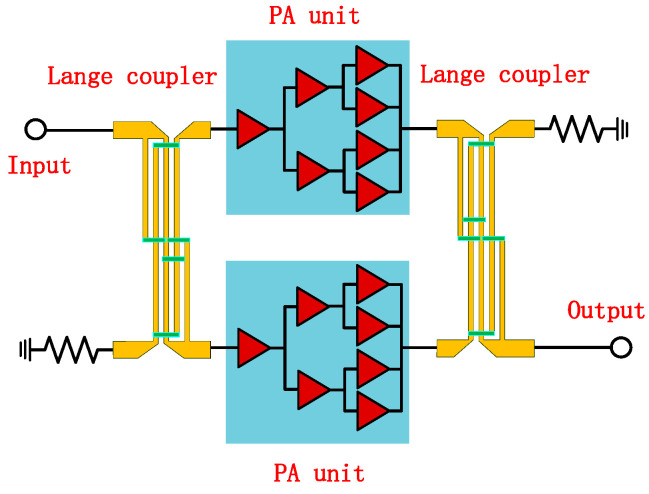
Topology of the balanced power amplifier architecture.

The designed W-band power amplifier operates over the frequency range of 80–86 GHz, delivering a linear gain greater than 17 dB, output power exceeding 35 dBm, and a (PAE) above 20%. To achieve the required high output power, the final stage of each amplifier unit employs eight parallel transistor cells for power combining. This multi-cell configuration not only ensures sufficient output capability but also effectively disperses heat sources, thereby reducing the thermal resistance of the chip and enhancing long-term reliability. To further improve performance, the amplifier adopts a fully balanced and symmetric topology, where two identical three-stage amplifier units are connected via Lange couplers at both the input and output ports for power splitting and combining. While cascading multiple gain stages is necessary to achieve the desired gain, this approach also increases power consumption and may compromise circuit stability. After comprehensive consideration, the adopted topology, as illustrated in Figure 2, uses two identical three-stage amplifier chains followed by balanced power combining.

Among various amplifier topologies, the balanced amplifier architecture is particularly advantageous for power combining, input/output impedance matching, and enhancing overall circuit stability. In this work, Lange couplers are utilized to implement the balanced structure. One additional benefit of the balanced configuration is that any reflection caused by a mismatch in the amplifier units is coupled back toward the input port with the opposite phase, resulting in cancelation of the reflected wave and thus improved return loss. For high-frequency applications, the efficiency of a balanced amplifier largely depends on the performance of the directional coupler, which requires low insertion loss and a wide coupling bandwidth.

### 3.1. Power Amplifier Design

Compared to devices operating at lower frequencies, active devices at millimeter-wave frequencies exhibit higher transmission losses and more pronounced phase imbalance among gate fingers. Therefore, to mitigate the degradation of circuit performance caused by these effects, the gate width of each finger should be limited, and the number of fingers should be carefully controlled. Considering the required output power, the insertion loss of the output matching network, and design margin—along with the process’s power density of 3.81 W/mm—a suitable total gate width for the output stage is determined. Larger total gate width enables higher output power, but at the cost of reduced gain. Thus, multistage amplification is necessary. In a multistage amplifier, the preceding stage must provide sufficient drive power for the following stage to ensure proper operation without significant compression. Typically, smaller-gate devices in the input stage provide sufficient gain, while larger-gate devices in the output stage ensure the desired output power. Therefore, selecting an appropriate gate width ratio between stages is crucial. In this work, a gate width ratio of 1:2:4 is adopted for the input stage, second stage, and output stage, respectively. The output stage has a total gate width of 1120 μm, which ensures both high gain and high output power. To mitigate the parasitic effects at W-band frequencies, each stage employs four-finger devices with a unit gate width of 35 μm. Each amplifier core adopts a multi-cell parallel configuration to synthesize power, and two identical amplifier cores are combined through Lange couplers. This structure effectively disperses thermal sources and enhances long-term reliability.

**Figure 3:**
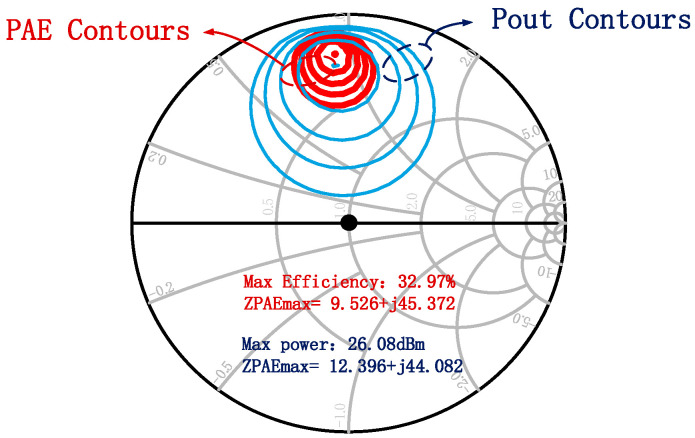
Load-pull results of a 4 × 35 μm active device.

The efficiency of the power amplifier is primarily determined by the loss of the output matching network. Therefore, the design starts with the output matching network, which transforms the output impedance of the active device to the standard 50 Ω at the output port. Figure 3 illustrates the PAE contours and Pout contours derived from load-pull simulations for a 4 × 35 μm HEMT at 84 GHz, as well as the optimal impedances for maximum efficiency and maximum output power. As shown, the optimal impedance for efficiency and that for output power do not coincide. Consequently, a trade-off must be made in the matching network design to balance efficiency and output power. A design prioritizing efficiency may sacrifice some output power, and vice versa. Similarly to the output stage, the input and inter-stage matching networks are designed to match the input impedance or the output impedance of the preceding stage to the input impedance of the subsequent stage. However, unlike the output stage, these matching networks primarily aim to optimize gain flatness and amplifier stability.

At W-band frequencies, conventional low-pass matching networks composed of lumped capacitors and inductors—commonly used at lower frequencies—become impractical due to their extremely small physical dimensions and significant losses. As a result, lumped elements are generally unsuitable for circuit design at W-band. Instead, high- and low-impedance transmission lines are favored due to their lower losses, ease of fabrication, and better process control. In this W-band power amplifier design, quarter-wavelength impedance transformers composed of alternating high- and low-impedance microstrip lines are employed for impedance matching, with series capacitors used to isolate the DC bias networks between stages. The DC-blocking capacitors and the bypass capacitors in the gate and drain bias networks are implemented using MIM capacitors, as they offer large capacitance values. The bias lines themselves are realized with quarter-wavelength high-impedance microstrip lines. Passive components are critical to the overall performance of the W-band power amplifier. To suppress parasitic oscillations and improve circuit stability, each gate bias line includes an RC branch consisting of a thin-film resistor and a shunt capacitor to ground.

**Figure 4:**
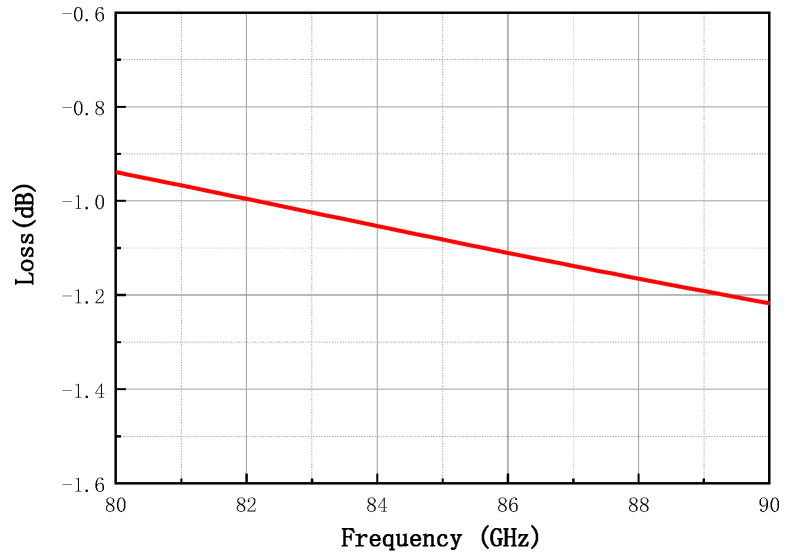
Simulated insertion loss in the output matching network.

To maximize amplifier efficiency, the insertion loss of the output matching network must be minimized. Therefore, low-loss, low-impedance transmission lines are used in the output matching network. Figure 4 shows the simulated insertion loss of the output matching network, which ranges from 0.9 dB to 1.2 dB across the 80–86 GHz band—ensuring high efficiency and high output power for the amplifier. Additionally, to maintain flat gain over the desired bandwidth, it is necessary to compensate for the inherent frequency-dependent gain degradation of the device. Typically, the gain of an active device decreases by approximately 6 dB per octave (as shown in Figure 1c). To address this, the inter-stage matching networks are designed to exhibit frequency-dependent loss with a negative slope (i.e., higher loss at lower frequencies and lower loss at higher frequencies), effectively compensating for the gain roll-off and resulting in a flat gain response across the operational bandwidth.

### 3.2. Lange Coupler Design

**Figure 5:**
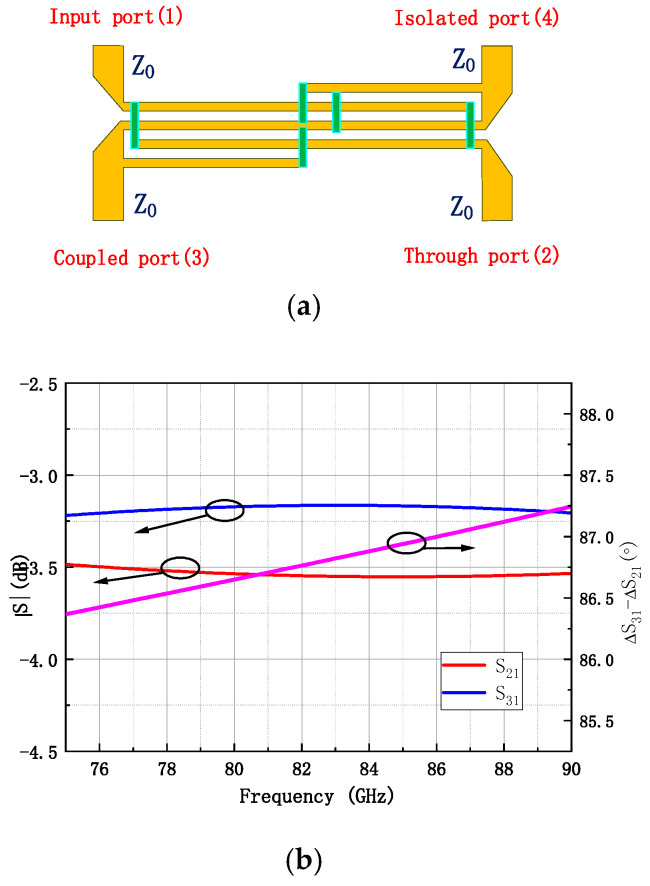
Lange Coupler. (**a**) Structure diagram (**b**) Simulated amplitude and phase values between ports.

Once the amplifier unit design is completed, a combining network is required to integrate the output power of the two amplifier units. In this work, a Lange coupler is employed for power combining. Figure 5a shows the structure of the Lange coupler, which consists of four ports: the input port, the through port, the coupled port, and the isolated port. Ideally, the amplitude at the coupled port is equal to that at the through port, with a 90° phase difference. The isolated port is connected to a 50-ohm resistor to ground. Figure 5b shows the simulated S-parameters of the Lange coupler. Over the 80–86 GHz frequency range, the amplitude imbalance and phase imbalance are within 0.5 dB and 3.5°, respectively.

**Figure 6:**
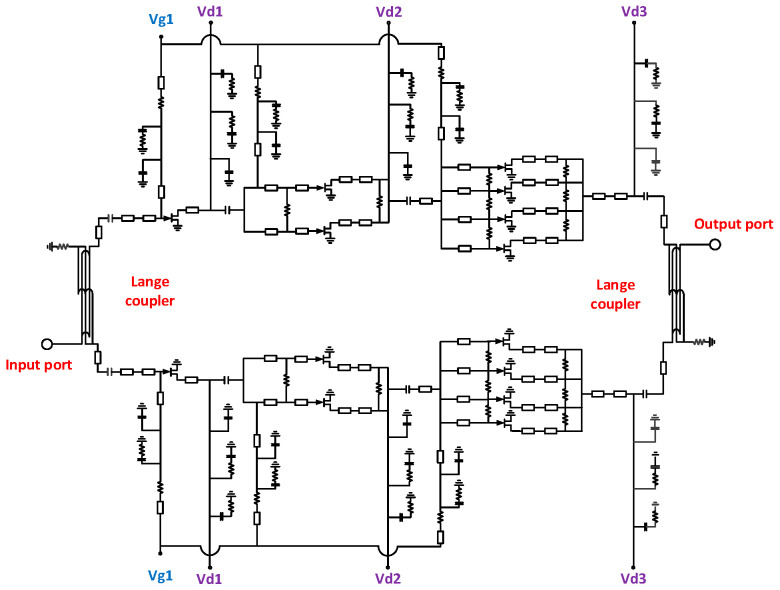
Schematic diagram of the W-band power amplifier.

Once both the Lange coupler and the power amplifier unit are designed, they are cascaded according to the structure shown in Figure 2 for further overall optimization. The schematic diagram of the cascaded amplifier is shown in Figure 6. Due to the high operating frequency of this W-band power amplifier, there are complex parasitic effects, and significant couplings between transmission lines. Therefore, full-wave electromagnetic field simulation techniques are employed to simulate the circuit layout. Based on the results from the electromagnetic simulation, the circuit structure is adjusted and iteratively optimized until the desired performance criteria are met. The final design is a high-efficiency, high-power W-band power amplifier with a compact chip size of 2.65 × 3.75 mm^2^.

## 4. Measurement Results

**Figure 7:**
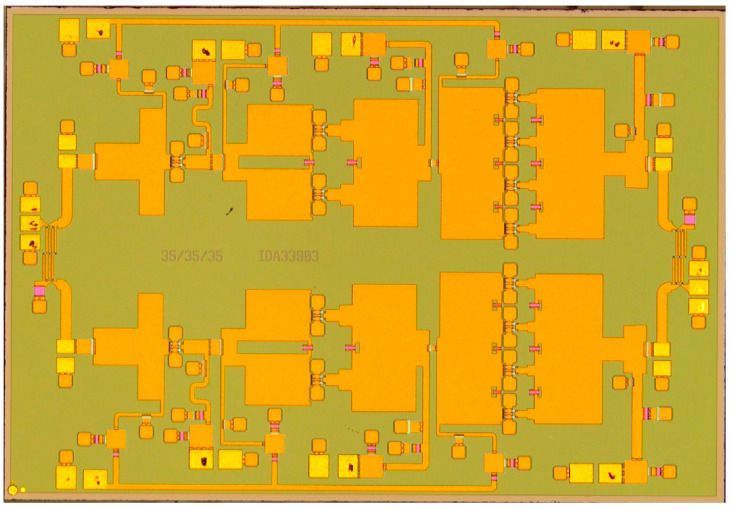
Photograph of the 80–86 GHz GaN high-efficiency power amplifier MMIC.

**Figure 8:**
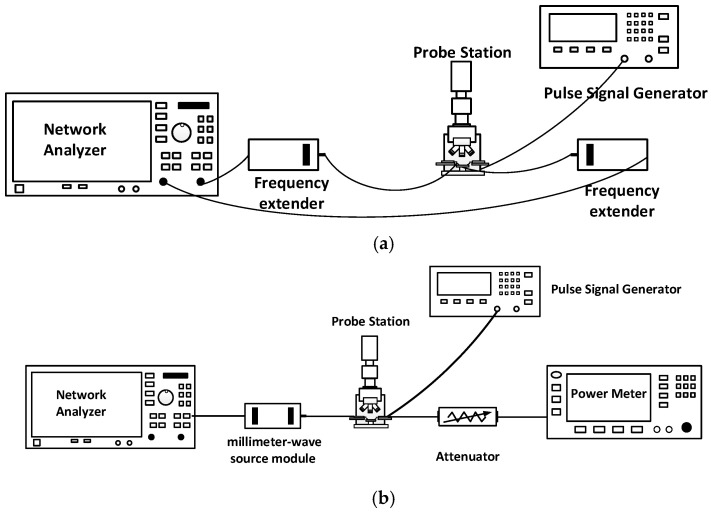
Measurement setup block diagrams: (**a**) small-signal, (**b**) large-signal.

**Figure 9:**
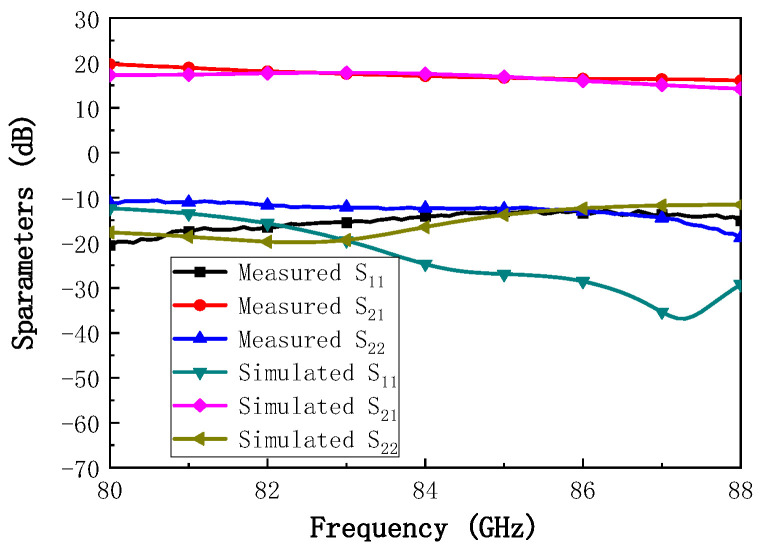
Small-signal S-parameters of the GaN power amplifier MMIC.

**Figure 10:**
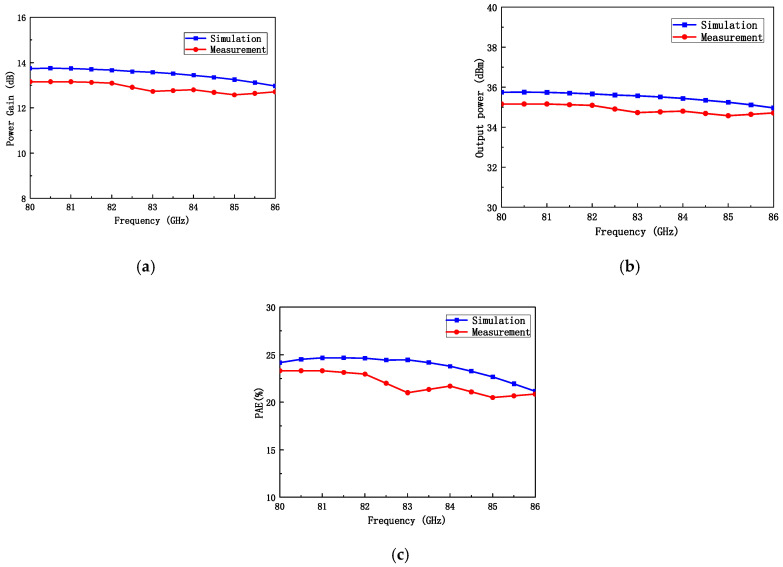
Power characteristics test results of the GaN power amplifier MMIC: (**a**) Power gain; (**b**) Output power; (**c**) Efficiency.

The chip fabricated using the 130 nm GaN process is shown in Figure 7. To characterize the RF performance of the proposed W-band power amplifier, small-signal S-parameters and large-signal power tests were conducted on the fabricated power amplifier chip. The measurement configurations are illustrated in Figure 8. For small-signal characterization (Figure 8a), on-wafer S-parameter measurements were performed using a probe station connected to a vector network analyzer (VNA) through frequency extenders, while pulsed biasing from a pulse signal generator was applied to the device under test (DUT) to suppress self-heating. For large-signal characterization (Figure 8b), the input signal was generated by a VNA, up-converted to the W-band via a millimeter-wave source module, and then fed into the DUT through the probe station. Pulsed drain biasing was provided to minimize thermal effects. At the output, an attenuator was inserted between the PA and the spectrum analyzer to reduce the signal level to within the measurable range and to protect the power meter from potential damage caused by high output power. The attenuated signal was subsequently recorded by the power meter for accurate power evaluation. Here, large-signal characterization was performed under pulsed conditions with a pulse width of 100 µs and a duty cycle of 20%. The test conditions were: Vd = +18 V, Vg = −2.2 V, and an excitation signal of −10 dBm. Pulsed operation was chosen to minimize excessive self-heating during on-wafer probing and to comply with the RF probe’s power handling limitations, which are only 1–2 W at W-band. Under these operating conditions, the amplifier was biased at the AB class operating point. The small-signal test results for the balanced amplifier are shown in Figure 9. The measured small-signal gain is greater than 17 dB in the 80–86 GHz range, which shows good consistency with the simulation values. The power characteristics of the power amplifier were tested under the same static bias conditions. During the power testing, the excitation signal power was 22 dBm. The power test characteristics are shown in Figure 10. From the figure, it can be seen that the power gain is greater than 12.5 dB in the 80–86 GHz range, with the saturation output power exceeding 35 dBm. The power-added efficiency (PAE) is above 20% within the operating range, with a peak value of 24%. The simulation results indicate an output power of approximately 38 dBm, while the measured value is slightly lower, around 35–36 dBm. This deviation can be attributed to several factors: (i) process variations between the fabricated devices and the HEMT models, (ii) minor inaccuracies in the EM modeling of passive components, and (iii) additional losses introduced by probing and the measurement setup. Such discrepancies between simulation and measurement are commonly observed in millimeter-wave GaN MMIC design and remain within an acceptable range.

Table 1 compares the performance of the design in this paper with that of advanced W-band power amplifiers reported in the current literature. The work reported in Reference [8] achieved a power-added efficiency of 21%, but the output power was relatively low. Another notable design is the work reported in Reference [9], which produced a peak output power of 35.5 dBm but with lower efficiency. The W-band power MMIC design in this paper achieves both high output power and efficiency, reaching an advanced level among similar designs both domestically and internationally, and stands out in the reported literature.

## 5. Conclusions

In this paper, a W-band balanced power amplifier is designed using an advanced 130 nm AlGaN/GaN HEMT process. The three-stage power amplifier achieves a linear gain greater than 17 dB, output power greater than 35 dBm, and an PAE of 24% within the 80–86 GHz frequency range. The W-band amplifier designed in this paper outperforms existing reported works in both output power and power-added efficiency. This demonstrates that the use of advanced GaN technology has great potential in improving the absolute power level and efficiency of W-band MMICs, paving the way for the enhancement of W-band power amplifier performance.

## Figures and Tables

**Table 1:** Comparison of power amplifier performance reported in other literature.

References	Process	Frequency (GHz)	Saturated Output Power (dBm)	Gain (dB)	PAE/%
[6]	GaN	75–110	25	22	14 (peak)
[7]	GaN	84–92	29.3	19.6	15 (peak)
[8]	GaN	92–96	32	10	20 (peak)/17.8 (avg.)
[9]	GaN	75–100	34	14	12 (peak)
[10]	GaN	90–100	18.3	8.2	7 (peak)
[11]	GaN	90–97	32.2	16.7	21 (peak)
[12]	GaN	86–94	30.6	12	8 (peak)
[13]	GaN	70–110	28.6	13	8.6 (peak)
This work	GaN	80–86	35	17	24 (peak)/22 (avg.)

avg. = average.

## Data Availability

The data are contained within this article.
